# *In vivo* data: treatment with the F11R/JAM-A peptide 4D decreases mortality and reduces the generation of atherosclerotic plaques in ApoE-deficient mice

**DOI:** 10.1016/j.dib.2020.105516

**Published:** 2020-04-23

**Authors:** Anna Babinska, Cristina C. Clement, Yan Li, Joanna Wzorek, Tomasz Przygodzki, Marcin Talar, Marcin Braun, Maria Swiatkowska, Yigal H. Ehrlich, Elizabeth Kornecki, Cezary Watala, Moro O. Salifu

**Affiliations:** a Department of Medicine, State University of New York, Downstate Medical Center, Brooklyn, New York 11203, USA; b Department of Pathology, Albert Einstein College of Medicine, New York 10461, USA; c Department of Neurology, State University of New York, Downstate Medical Center, Brooklyn, New York 11203, USA; d Department of Haemostasis and Haemostatic Disorders, Biomedical Sciences, Medical University of Lodz, 92-215 Lodz, Poland; e Department of Pathology, Medical Univeristy of Lodz, 92-213 Lodz, Poland; f Department of Cytobiology and Proteomics, Biomedical Sciences, Medical University of Lodz, 92-215 Lodz, Poland; g Program in Neuroscience, College of Staten Island of the City University of New York, Staten Island, New York 10314, USA; h Department of Cell Biology, State University of New York, Downstate Medical Center, Brooklyn, New York, 11203, USA

**Keywords:** ApoE**^-/-^**mice, Atherosclerosis, Endothelium, F11R, F11R/JAM-A, Inflammation, F11R Peptide 4D, Platelet F11 Receptor, Severe skin lesion

## Abstract

The data in this article focus on the F11 Receptor (F11R/JAM-A; Junctional Adhesion Molecule-A; JAM-A, F11R), a cell adhesion protein constitutively expressed on the membrane surface of circulating platelets and localized within the tight junctions of healthy endothelial cells (ECs). Previous reports have shown that F11R/JAM-A plays a critical role in the adhesion of platelets to an inflamed endothelium due to its’ pathological expression on the luminal surface of the cytokine-inflamed endothelium. Since platelet adhesion to an inflamed endothelium is an early step in the development of atherosclerotic plaque formation, and with time, resulting in heart attacks and stroke, we conducted a long-term, study utilizing the atherosclerosis-prone ApoE**^-/-^** mice to attempt a blockade of the formation of atherosclerotic plaques by preventing the adhesion of platelets to the inflamed vasculature *in vivo*. Utilizing a nonhydrolyzable peptide derived from an amino acid sequence of F11R/JAM-A, peptide 4D, we have shown *in culture* that the adhesion of platelets to the inflamed endothelial cells could be blocked by peptide 4D. The present data demonstrate the positive health benefits of chronic peptide 4D administration to the atherosclerosis-prone ApoE^-/-^ mice, and provides new information for potential use of this F11R derived peptide in the prevention of atherosclerosis. The data presented in this article provide further experimental support for the study presented in Babinska et al., Atherosclerosis 284 (2019) 92-101.

Specifications Table

**Table:** 

Subject	Cardiology and Cardiovascular Medicine
Specific subject area	Atherosclerosis
Type of data	Table
	Images
	Graphs
	Figures
	Videos
How data was acquired	Observation
	Mouse model ApoE^-/-^
	Microscope
	Pathology slides
	Softwares: Image J and FIJI
Data format	Raw
	Analyzed
Parameters for data collection	Animal facilities at SUNY Downstate Medical Center: treatment of ApoE^-/-^ mice with peptide 4D or saline and observation for wellness and survival. Basic Research Laboratory at SUNY: tissue collection and analysis of plaque size in the aorta and heart using Image J software. Biomedical Sciences, Medical University of Lodz, Poland: analysis of effect of peptides on platelet adhesion to endothelium using intravital microscopy with TrackMate plugin and FIJI software.
Description of data collection	*In vivo* experimental ApoE^-/-^ mice data *COLLECTION,* Sections obtained from the aorta and heart of each animal were stained with H&E. Images were captured with a microscope (Nikon) equipped with a color video camera (Motic Images Plus 2.0) attached to a computerized imaging system. Lesion areas or necrotic areas where measured and summarized using software Image-J and data were collected on the computer using Excel program. Platelets’ tethering to vascular wall was recorded in mice with the use AxioExaminer microscope (Zeiss) equipped with Rolera EM-C2 camera (Bioimaging Solutions). Platelets’ motility was analysed with TrackMate plugin implemented in FIJI software.
Data source location	SUNY/Downstate Medical Center, Brooklyn, New York;
	Biomedical Sciences, Medical University of Lodz, Lodz Poland.
Data accessibility	With the article
Related research article	A. Babinska, C.C. Clement, T. Przygodzki, M. Talar, Y. Li, M. Braun, J. Wzorek, Maria Swiatkowska, Y. H. Ehrlich, E. Kornecki, C. Watala, M.O. Salifu.
	A peptide antagonist of the F11 Receptor (F11R; JAM-A) prolongs survival and reduces plaque formation in an animal model of atherosclerosis. Atherosclerosis 284 (2019) 92-101.

## Value of the data

• The data reported here deepen and broaden the effect of the chronic treatment of ApoE^-/-^ mice by the nonhydrolyzable F11R Peptide 4D.
• The new data reported here represent the first step in the development of a novel class of drugs, based on the sequence of F11R/JAM-A, to be used for the prevention and treatment of atherosclerosis.
  The data presented here, obtained *in vivo*, demonstrate the efficacy of F11R-based drugs and open up the way for conducting direct, clinical trials in patients with cardiovascular disease.
• The data reported here include video films that directly show the significant blockade of the adhesion of platelets onto the inflamed endothelium in the presence of the F11R peptide 4D and thus amplify and strongly support the dataset in Babinska et al., Atherosclerosis, 284 (2019) 92-101.

## 1 Data Description

**Fig. 1:**
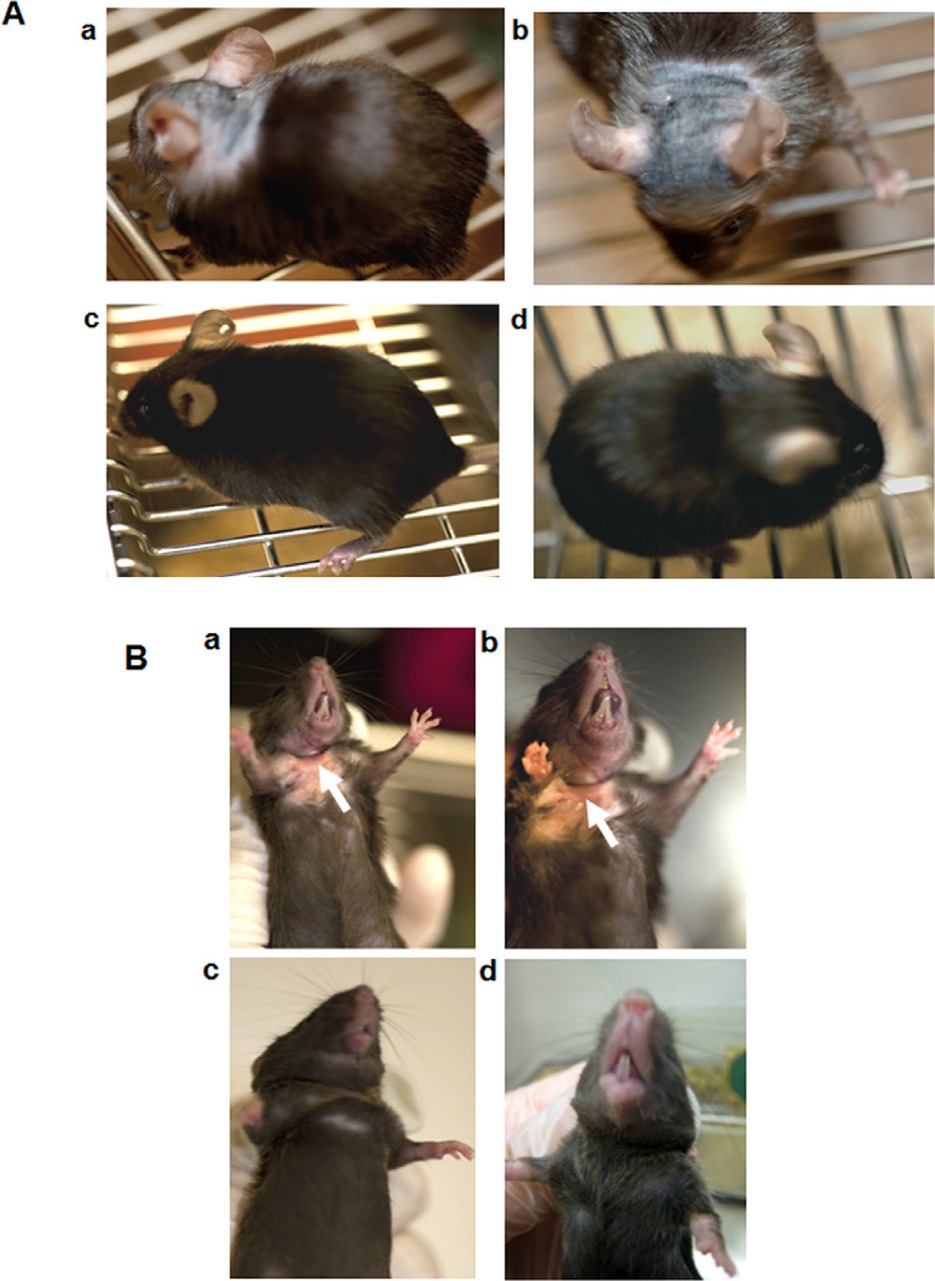
Comparative data of the overall heathy external physical appearance of ApoE^-/-^ mice treated with peptide 4D for 3 months, versus the unhealthy appearance of the untreated control ApoE^-/-^ mice administer only the saline (vehicle) during the same period of time. Fig. 1A. Panels: a and b. The unhealthy physical appearance of control, untreated ApoE^-/-^ mice injected daily, intraperitoneally, with the 200µl saline (vehicle); injections were initiated at six-weeks of age. Fig. 1A. Panels: c and d. The healthy physical appearance of peptide 4D treated ApoE^-/-^ mice, treated daily by intraperitoneal injections of peptide 4D; injections were initiated at six-weeks of age. Fig. 1B. Panels: a and b. The severe skin lesion on the chest of control, untreated ApoE^-/-^ mice. Mice were injected intraperitoneally with the saline (vehicle) for a 3 month period. Injections were initiated at six-weeks of age. The white arrows point to the severe skin lesion/scratching observed on the chests of control mice. Fig. 1B. Panels: c and d. The healthy appearance of the skin on the chests of peptide 4D treated ApoE**^-/-^** mice. ApoE^-/-^ mice were treated with peptide 4D for a 3 month period, with daily intraperitoneal injections initiated at six-week of age**.** Photographs are representative of two mice obtained from either the control group or the peptide 4D treated group of ApoE^-/-^ mice.

**Fig. 2:**
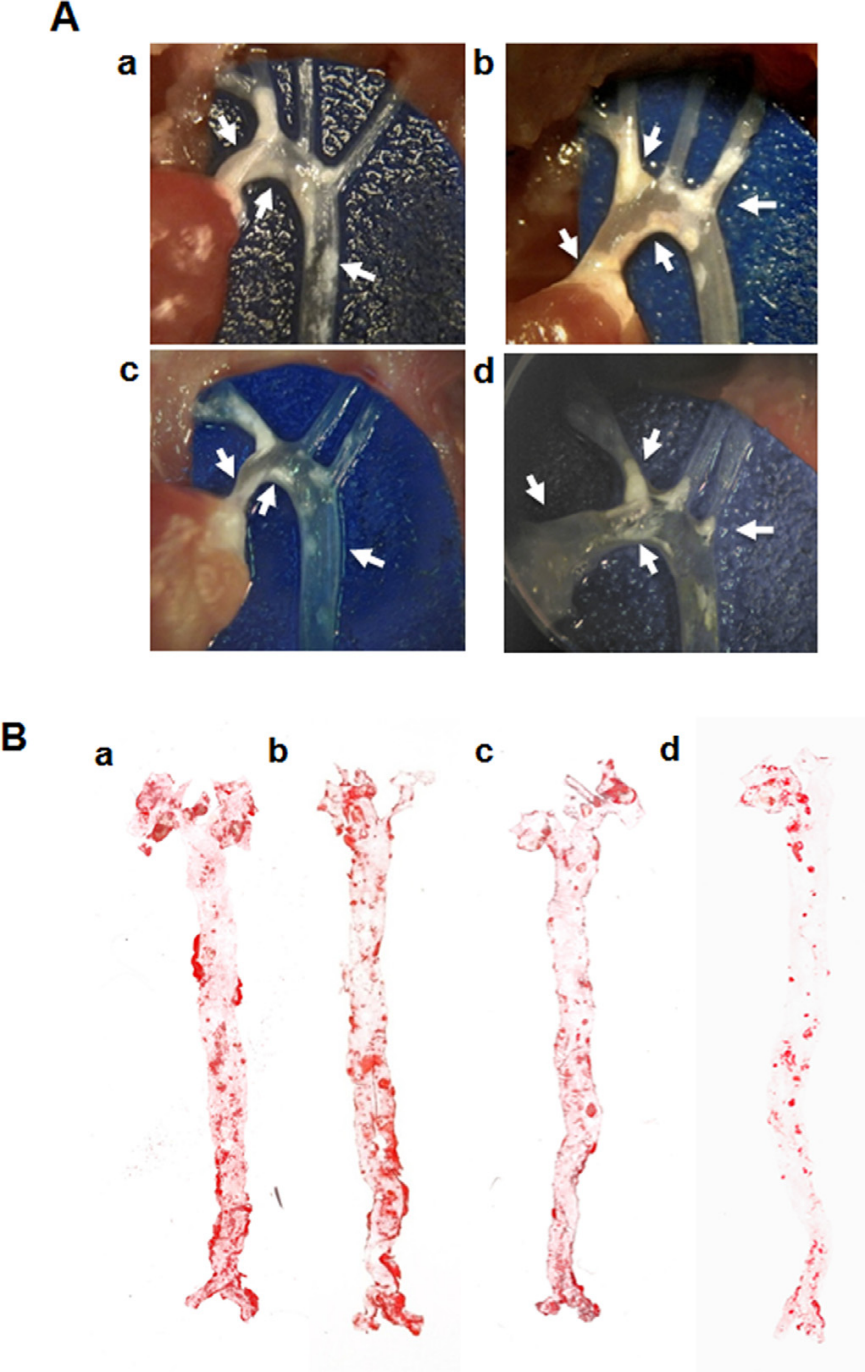
Examination of the development of atherosclerotic plaques in the aortic arch and whole aorta of ApoE^-/-^ mice treated with peptide 4D in comparison to plaques developed in blood vessels of untreated control ApoE^-/-^ mice. Fig. 2A. Panels: a and b. Control, untreated mice were injected with the saline (vehicle) for a period of 3 months. The arrows point out the accumulation of atherosclerotic plaques in the ascending aorta, the aortic arch, the brachiocephalic artery, the left subclavian artery, and the proximal portion of the descending aorta. The photographs are representative of two animals obtained from the control group of mice. Fig. 2A. Panels: c and d. Photographs of the aortic arches the peptide 4D-treated ApoE^-/-^ mice. The arrows point to comparative areas of the ascending aorta, the aortic arch, the brachiocephalic artery, the left subclavian artery, and the proximal portion of the descending aorta observed in the peptide 4D treated mice. The photographs are representative of two animals obtained from the peptide 4D treated group of mice. Fig. 2B. Panels: a and b. Control; Photographs of Oil Red O staining of the whole aortas of ApoE^-/-^ mice injected with saline for a 3 month period. The photographs depict the staining pattern obtained from two control mice. Fig. 2B. Panels: c and d. Peptide 4D-treated; Photographs of Oil Red O staining of the whole aortas of ApoE^-/-^ mice treated with peptide 4D for a 3 month period. Photographs represent two mice obtained from the peptide 4D- treated group.

**Fig. 3:**
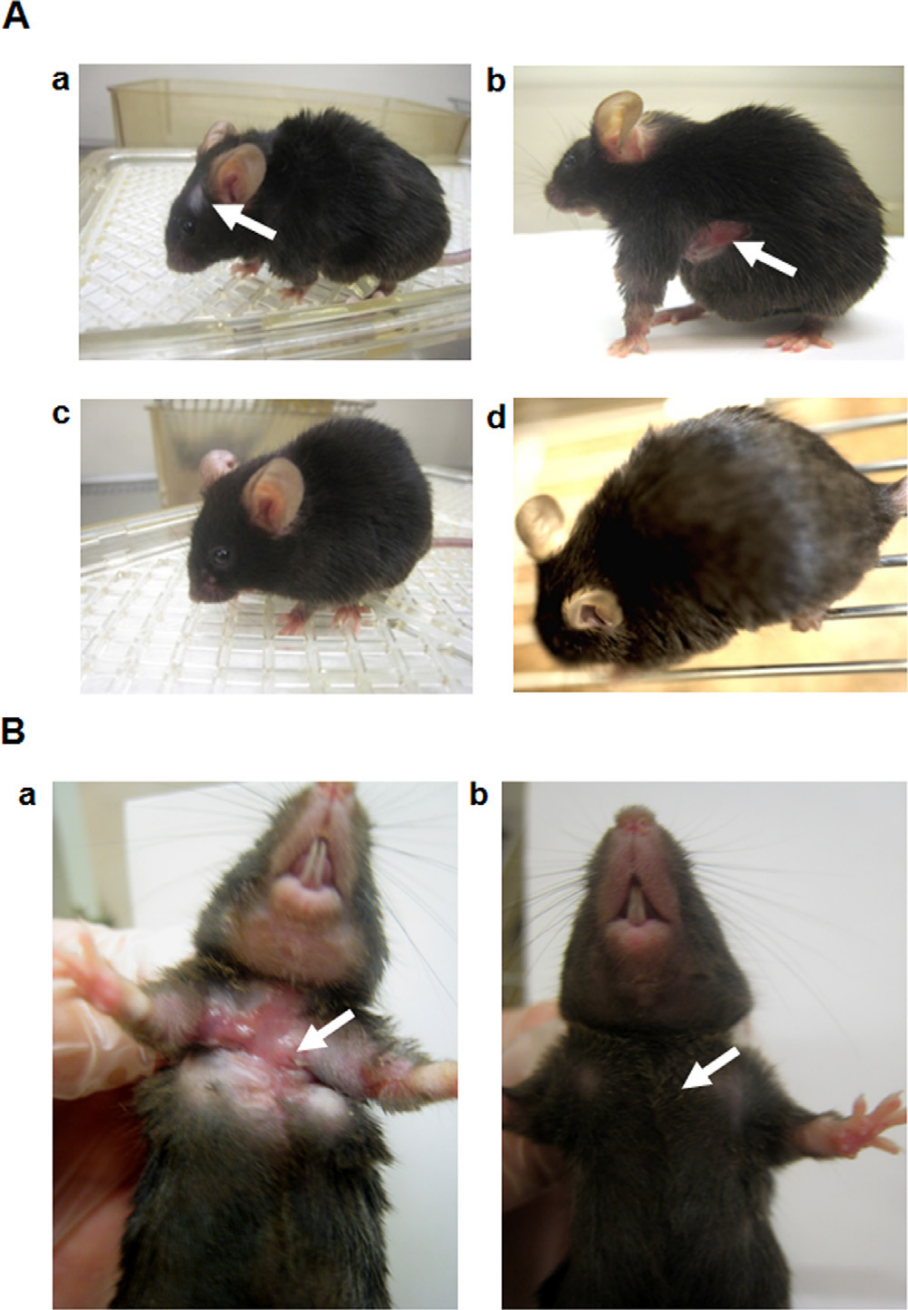
Survivability and early treatment with peptide 4D. The effects of early, continuous treatment of ApoE^-/-^ mice with peptide 4D on survivability were examined. The physical appearance of 10 ½ months old ApoE^-/-^ mice previously treated with peptide 4D for a 4 month period of time were compared to control untreated mice. Fig. 3A. Panels: a and b. ApoE^-/-^ mice were injected intraperitoneally with the saline vehicle for a 4 month period of time, and afterwards, saline injections were terminated, and the mice were observed at 10 ½ month of age. The white arrows point to lesions observed on the skin of control untreated mice. Fig. 3A. Panels: c and d. ApoE^-/-^ mice were injected intraperitoneally daily with peptide 4D for a 4 month period of time, and afterwards, these injections were terminated and the mice were observed at 10 ½ month of age period. Fig. 3B. Panel a. Control, vehicle-treated ApoE^-/-^mouse showing severe skin lesion (arrow) on the chest. The mice were observed at 10 ½ month of age. Photograph represents one of two mice. Fig. 3B. Panel b. Peptide 4D treated-ApoE^-/-^ mice were initially treated with peptide 4D daily for a 4 month period of time, and afterwards, the injections were terminated and animals were observed at 10^1^/_2_ month of age. The arrow shows the lack of lesion in the mouse following an early, 4 month treatment period with peptide 4D. Photograph represents one of two mice.

**Fig. 4:**
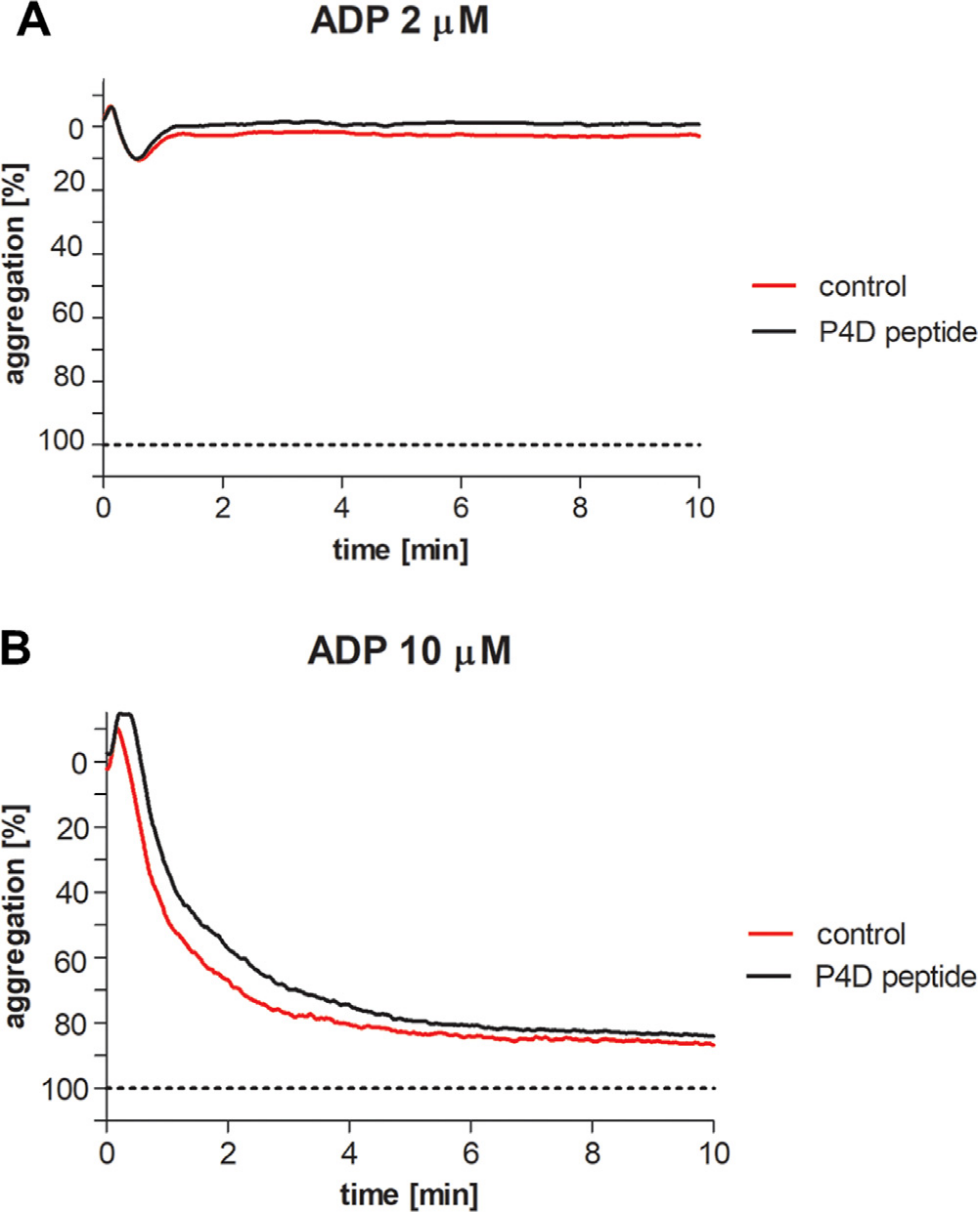
Test of the effect of peptide 4D on the ADP-induced aggregation of human platelets (in platelet-rich plasma). Fig. 4A. This figure depicts a representative tracing of platelet aggregation induced by ADP (2µM) in the presence of peptide 4D (P4D; 500µM, final concentration), in comparison to the ADP-induced platelet aggregation in the absence of peptide 4D (control). Fig. 4B. This figure depicts a representative tracing of platelet aggregation induced by ADP (10µM) in the presence of peptide 4D (P4D, 500µM final concentration), in comparison to the ADP-induced platelet aggregation in the absence of peptide 4D (control).

**Fig. 5:**
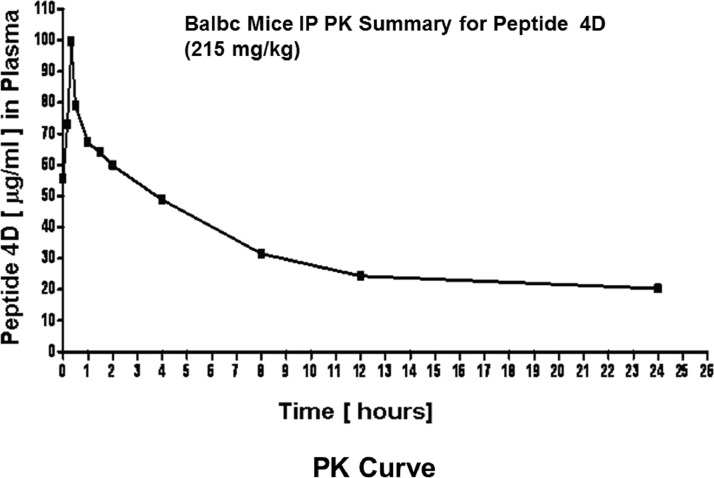
Pharmacokinetic (PK) data of Peptide 4D. Figure 5 shows the PK data for peptide 4D, as vs time curves, thereby allowing for the determination of the PK parameters analyzed by LC-MS-MS. The concentration of peptide 4D was measured in the plasma at various time points, and is expressed in µg/ml. The half-life of peptide 4D was measured to be over 3.5 hours. Time points of 15 min, followed by 1, 2, 4, 5, and 24 hours after the administration of peptide 4D were selected to generate the concentration of Cmax, Tmax, area under the plasma concentration time curve (AUC), drug clearance, terminal elimination half-life, and volume of distribution.

The F11 Receptor (F11R/JAM-A; Junctional Adhesion Molecule-A; JAM-A; F11R) is a cell adhesion protein present on the surface of circulating platelets and located within the tight junctions of endothelial and epithelial cells. The data presented here confirm *in-vivo* evidence for the critical role of F11R/JAM-A in the formation of atherosclerotic plaques in ApoE^-/-^ mice and provide further strong experimental support for our work in Babinska et al. (2019) [1]. The data demonstrate that within a 3-month period of daily administration of either peptide 4D (group 1) or the vehicle control (group 2) to ApoE^-/-^ mice, two of the mice in the control group already were unable to continue the study as assessed by their extremely unhealthy physical appearance. These mice were euthanized, and at the same time, two mice obtained from the peptide 4D-treated group were randomly-selected and euthanized, and their tissues examined histologically in comparison to the controls. The data demonstrate that differences were observed in the overall physical appearance and degree of atherosclerotic plaques detected in the major blood vessels of the peptide 4D treated mice and the untreated control mice (data shown in Figs. 1 through 3). The data shown in Fig. 1 demonstrate that the external healthy appearance of ApoE^-/-^ mice treated with peptide 4D for a three month period differs significantly from the unhealthy physical appearance of untreated control ApoE^-/-^ mice. The data shown in Fig. 2 indicate the presence of large accumulation of atherosclerotic plaque as observed in the aortic arch and the whole aorta of the control, untreated ApoE^-/-^ mice, whereas, mice treated with peptide 4D showed a much diminished plaque accumulation in the same blood vessels. The data presented in Fig. 3, compare the overall health and physical appearance of 10^1^/_2_ months old ApoE^-/-^ mice following their termination of a 4- month injection period with either peptide 4D or the vehicle. The data in Fig. 4 and Table 1 demonstrate the lack of an inhibitory effect by peptide 4D on ADP-induced platelet aggregation. Data of Fig. 5 display the pharmacokinetic analysis (PK) of peptide 4D *in vivo*. Data display by videos: videos depicting platelet adhesion to the walls of blood vessels using the Intravital Microscopy System are provided and available in this article as the Supplemental Files. Data: Videos 1a and 1b display platelet adhesion to the arteriole in the presence of peptide 4D; Data: Videos 2a and 2b display the arteriole in the presence of scrambled peptide, control (scrambled F11R peptide 4D); Data: Videos 3a and3b display platelet adhesion to the venule in the presence of peptide 4D; Data: Videos 4a and 4b display platelet adhesion to the venule in the presence of scrambled peptide (control).

**Table 1:** Examination of three concentrations of peptide 4D (P4D) on ADP-induced platelet aggregation. The data depict the maximal platelet aggregation [%] measured, in response to 10µM ADP in the presence of peptide 4D (P4D) or in the absence of the peptide (control). Values shown are the Means ± SD; n=3-7. Paired Student's t- test was used for statistical analysis; significance *P*<0.05.

	ADP concentration	
	10 µM	
P4D peptide concentration [µM]	P4D peptide	Control
100	70 ± 12	71 ± 11
250	73 ± 13	75 ± 9
500	74 ± 20	79 ± 10

## 2 Experimental design, materials and methods

### 2.1 Pharmacokinetic (PK) data obtained with Peptide 4D

Drug pharmacokinetics (PK) *in vivo* is a key requirement for the evaluation of the stability and availability of peptide 4D, as a potential compound for use in the next phase of our investigations. Accordingly, PK data of peptide 4D were obtained by the Services of Bio Quant, San Diego, CA. Thus far, a single dose of the F11R peptide 4D and a time perfusion course were performed in Balb/c mice. Time points of 15 min, followed by 1, 2, 4, 5, and 24 hr after the administration of peptide 4D to animals, were selected to generate concentration vs time curves thereby allowing for the determination of the PK parameters of maximal concentration (Cmax), maximal time (Tmax), area under the plasma concentration time curve (AUC), drug clearance, terminal elimination half-life, and volume of distribution of peptide 4D. The PK data of peptide 4D performed by LC-MS-MS; units of measurement (µg/ml), indicated a half-life of peptide 4D of over 3.5 hours *in vivo*. In this regard it is known that peptides, based on drugs approved by the FDA or in human clinical trials such as A6 peptide or Fuzeon from Roche, have a half-life in blood of about 2-3 hrs making the half-life of the F11R peptide 4D very desirable for utilization in vivo. Peptide 4D is rapidly available in plasma with a Tmax of 0.3 hours. Thus, PK data demonstrate that peptide 4D has sufficient half-life in the circulation to be evaluated in the ApoE^-/-^ animal model of atherosclerosis.

### 2.2 Dosage and frequency of administration of Peptide 4D *in vivo*

The dosage calculated for the administration of peptide 4D *in vivo* was selected as the concentration shown to completely inhibit the stimulatory monoclonal antibody M.Ab.F11-induced platelet aggregation *in vitro,* and the dosage is based also on the PK data obtained *in vivo.* Data obtained from dose-response showed that peptide 4D (0.3 mg) (670 µg/ml) completely inhibited M.Ab.F11-induced platelet aggregation *in vitro*. Based on these data with M.Ab.F11, and taking into account the mouse extracellular volume of 6.44 ml, we calculated that a comparable dose of peptide 4D, of 215 mg/kg body weight/mouse would be needed, as the amount of peptide 4D to be utilized here. Based on these findings, we performed pharmacokinetic (PK) studies in Balb/c mice to determine the half-life and final concentrations of peptide 4D in plasma. Based on the PK data of peptide 4D, the dose that was used for animal injections, was calculated as 215 mg/kg, and administered, long-term, to ApoE^-/-^ mice by daily intraperitoneal injections.

### 2.3 Ethics statement

The Animal Care and Use Committee of SUNY Downstate Medical Center approved this work. Experiments involving all ApoE^-/-^ mice (approval number 10-337-09).

For the intravital microscopic experiments the studies were approved by the Local Ethical Committee on Animal Experiments, Medical University of Lodz (approval number 65/LB572/2011).

This research was performed in strict accordance with the recommendation of the Fundamental Guidelines for Proper Conduct of Animal Experiment.

### 2.4 ApoE^**-/-**^ mice, diet and injection regimen

Female Apo E^-/-^ mice (six weeks old) of a C57BL/6J background, (Jackson Laboratory, Bar Harbor, Me), were divided into two separately-housed groups. Group 1 was treated with peptide 4D by daily, intraperitoneal injections with peptide 4D (4.3 mg/200µl) for a 3 month period. Group 2 was injected daily with an equivalent amount of diluent vehicle (200µl of 0.9% saline) for the same 3- month period. All animals were fed a regular Rodent Diet (Calories provided by protein 28.1%, fat 12.13% and carbohydrates 59.8%) for two months, then the diet was switched to a Western-type diet (TD.88137 Adjusted Calories Diet (42% from fat) (0.15% cholesterol, 21% anhydrous milk fat) (Harlan Laboratories Inc.) to induce hypercholesterolemia for a period of one month [1], [2]. Following 3 month of injections with either the F11R peptide 4D or the vehicle, saline, the animals were euthanized. The aortas and hearts were isolated, dissected, and the atherosclerotic lesions were analyzed.

### 2.5 Dissection and examination of the aorta and hearts of ApoE^**-/-**^ mice for the presence of atherosclerotic lesions

Following euthanasia, the aortas were prepared for dissection and the aortic arches were photographed. Following dissection*, en face* lesion surface area assay was performed on the aortas and hearts as previously described [3], [4], [5]. The dissected aortas were stained with Oil-Red-O stain modified from previously described methods [3], [4], [6]. For morphometric lesion analysis [3], [4], [5] sections from the heart of each animal were stained with Harris hematoxylin-eosin. The total intimal lesion area and the necrotic core area (NE) per cross section were quantified by taking the average of 6 sections spaced 30 µm apart, beginning at the base of the aortic root. The necrotic core areas, acellular/anuclear areas (negative for hematoxylin-positive nuclei, white areas of lesion) were measured per cross section from the same slides where total intimal lesion area was measured. Images were viewed and captured with a microscope (Nikon) equipped with a color video camera (Motic Images Plus 2.0) attached to a computerized imaging system. Lesion areas or necrotic areas (white areas of lesion) where measured and summarized using software Image-J. The aortas were fixed in PBS/4% formaldehyde for 3 days, washed in PBS followed by staining with 1.8% Oil-Red-O in 60% isopropanol for 15 min at 22°C, then de-stained in 60% isopropanol for 5 min. The stained aortas were placed on slides and stored at 4°C. Images of the whole aortas were captured with a Nikon digital camera and analysis of aortic lesion areas observed from the aortic root to the diaphragm, including the aortic arch, was achieved using the Image Analysis software Image-J.

### 2.6 Histological and immunohistological analysis

The tissue samples were fixed in 10% buffered formalin and embedded in paraffin (FFPE), and 5 µm-thick sections were stained with hematoxylin and eosin, Masson-trichrome or with different specific antibodies. The atherosclerotic plaques were examined by light microscopy and by using UltraFast Scanner (Philips IntelliSite Solution, USA) with DigiPath™ Professional Production Software (Xerox, Norwalk, CT, USA). For histochemistry and immunohistochemistry staining the following antibodies were used: anti-CD3 for T-lymphocytes (Polyclonal, A0452 (Dako, Agilent), anti-CD68 for macrophages (PG-M1, M0876, Dako, Agilent), anti-SMA for vascular smooth muscle cells (VSMC, clone 1A4, M0851, Dako, Agilent) and Masson-trichrome stain for collagen evaluation (according to routinely applied staining procedure). All antibodies display cross reactivity with mice according to manufacturer's and literature data [7], [8], [9]. A positive mice tissue control for each staining procedure was used. For immunohistochemical analysis monoclonal antibodies (FLEX Monoclonal Mouse Anti-Human, Ready-to-Use (Link), Dako, Agilent) and EnVisionTMFLEX + (Dako, Agilent) for the visualization were used. The tests were carried out using Autostainer Link 48 (Dako, Agilent).

### 2.7 Platelet aggregation

Platelet aggregation in platelet-rich plasma (PRP) was carried-out by using the turbidimetric method in a platelet aggregometer (Chronolog, Haverstown, PA) [10]. Aliquots of the PRP were maintained at 37°C. The effect of peptide 4D on platelet aggregation was examined by utilizing three concentrations of peptide 4D (final concentrations of 100µM, 250µM and 500 µM). The PRP was incubated for 2 minutes with peptide 4D prior to the addition of ADP at final concentrations of 2µM (subthreshold concentration) or 10µM, for the initiation of platelet aggregation.

## Appendix Supplementary materials

Image, application 1
